# Anticandidal Potential of Two Cyanobacteria-Synthesized Silver Nanoparticles: Effects on Growth, Cell Morphology, and Key Virulence Attributes of *Candida albicans*

**DOI:** 10.3390/pharmaceutics13101688

**Published:** 2021-10-15

**Authors:** Reham Samir Hamida, Mohamed Abdelaal Ali, Doaa A. Goda, Alya Redhwan

**Affiliations:** 1 Molecular Biology Unit, Department of Zoology, Faculty of Science, Alexandria University, Alexandria 21500, Egypt; reham.hussein@alexu.edu.eg; 2 Biotechnology Unit, Department of Plant Production, College of Food and Agriculture Science, King Saud University, Riyadh 12372, Saudi Arabia; mali3@ksu.edu.sa; 3 Bioprocess Development Department, Genetic Engineering and Biotechnology Research Institute (GEBRI), City of Scientific Research and Technological Applications (SRTA-City), Alexandria 21934, Egypt; doaa.rashid@yahoo.com; 4 Department of Health, College of Health and Rehabilitation Sciences, Princess Nourah bint Abdulrahman University, Riyadh 11564, Saudi Arabia

**Keywords:** silver nanoparticles, oxidative stress, ultrastructural alterations, *Hwp1*, *CDR1*

## Abstract

*Candida albicans* is an opportunistic human fungal pathogen responsible for 90–100% of mucosal and nosocomial infections worldwide. The emergence of drug-resistant strains has resulted in adverse consequences for human health, including numerous deaths. Consequently, there is an urgent need to identify and develop new antimicrobial drugs to counter these effects. Antimicrobial nanoagents have shown potent inhibitory activity against a number of pathogens through targeting their defense systems, such as biofilm formation. Here, we investigated the anticandidal activity of silver nanoparticles biosynthesized by the cyanobacterial strains *Desertifilum* sp. IPPAS B-1220 and *Nostoc* Bahar_M (D-SNPs and N-SNPs, respectively), along with that of silver nitrate (AgNO_3_), and examined the mechanisms underlying their lethal effects. For this, we performed agar well diffusion and enzyme activity assays (lactate dehydrogenase, adenosine triphosphatase, glutathione peroxidase, and catalase) and undertook morphological examinations using transmission electron microscopy. The effects of the three treatments on *Hwp1* and *CDR1* gene expression and protein patterns were assessed using qRT-PCR and SDS–PAGE assays, respectively. All of the three treatments inhibited *C. albicans* growth; disrupted membrane integrity, metabolic function, and antioxidant activity; induced ultrastructural changes in the cell envelope; and disrupted cytoplasmic and nuclear contents. Of the three agents, D-SNPs showed the greatest biocidal activity against *C. albicans.* Additionally, the D-SNP treatment significantly reduced the gene expression of *Hwp1* and *CDR1*, suggestive of negative effects on biofilm formation ability and resistance potential of *C. albicans*, and promoted protein degradation. The mechanism involved in the biocidal effects of both D-SNPs and N-SNPs against *C. albicans* could be attributed to their ability to interfere with fungal cell structures and/or stimulate oxidative stress, enabling them to be used as a robust antimycotic agent.

## 1. Introduction

*Candida albicans*, a dimorphic yeast, is an opportunistic pathogen and the primary causative agent of candidiasis worldwide [1]. Although a commensal in the gastrointestinal, genitourinary, and respiratory tracts, *C. albicans* is a potential source of infection in immunocompromised individuals [2]. A reduction in pH in the above-mentioned tracts of a healthy individual can alter the composition of the microbiota, resulting in candidiasis. Additionally, its entry into the bloodstream during a systemic infection frequently causes “candidemia”, reaching mortality rates of up to 70% [3,4]. Treatment options for *Candida* infection include the polyenes amphotericin B and nystatin and the azoles fluconazole and itraconazole. However, these drugs elicit adverse effects, including nephrotoxicity, hepatotoxicity, red blood cell toxicity, and cardiotoxicity, which are attributed to their mechanism of action [4,5]. Recently, nanoantibiotics, comprising therapeutic or diagnostic/detection tool-based nanomaterials (NMs), have emerged as a novel paradigm for mitigating infectious diseases in the microbial multidrug-resistant era [6]. Many studies have shown that NMs act as potent antimicrobial agents against different microbial pathogens, including bacteria [7], fungi [8], and cyanobacteria [9]. Moreover, NMs have the potential to enhance and improve the therapeutic activity and reduce the side effects of many antimicrobial drugs, as well as alleviate microbial resistance to these treatments [6,10,11]. These antimicrobial activities of NMs are due to their physicochemical properties, including small size, large surface area, and surface charge, which enable them to easily invade tissues and adhere to microbial cell envelopes. This results in cell wall and cytoplasmic membrane disruption, a surge in membrane permeability, and interference with cellular biomolecules, leading to cellular dysfunction and apoptosis [6,12,13,14]. Despite their many advantages when compared with traditional antibiotics, including reduced acute toxicity, the ability to overcome microbial resistance, lower costs [6,11,15,16], as well as the biocompatibility of nanoparticles (NPs) and nanoantibiotics, particularly with long-term exposure, remains an overriding concern [17,18]. Green synthesis is an emerging concept that involves the use of eco-friendly approaches to synthesize NPs, thereby reducing their toxicity against the environment and living organisms [19,20]. This green route mimics the behavior of living organisms during the detoxification of heavy metals from their environment [21,22]. In the laboratory, NPs are fabricated from their precursors using natural sources such as plants, algae, lichens, yeast, and extracted biomolecules such as natural pigments, enzymes, proteins, and polysaccharides, etc. These organisms and their biomolecules represent sources of the reducing and stabilizing agents required for NP synthesis [19,21]. Among the metallic NPs, silver NPs (Ag-NPs) are the leading antimicrobial nanoagents due to their physicochemical properties and inhibitory activities against bacteria and fungi [23]. For instance, KerraContact Ag and Acticoat Flex 7, silver nanocrystal-based dressings, have been approved by the United States Food and Drug Administration for use as burn therapy [24,25,26]. Although the antimicrobial properties of Ag-NPs have been well documented, relatively few studies have focused on the anticandidal activity of Ag-NPs or explored their mechanism of action [18,27]. Nevertheless, there is consensus that the main mechanism of action underlying the anticandidal potential of Ag-NPs involves direct effects on the cell envelope, including the cell wall and membranes. This interference disrupts membrane integrity, leading to the formation of pores, holes, and folds, thus enabling NPs to penetrate fungal cells and promote the denaturation of many biomolecules, cytoplasmic dissolution, and genetic material damage. The mechanism underlying the lethal effects of Ag-NPs against *C. albicans* is thought to also involve their ability to promote reactive oxygen species (ROS) production, which causes extensive oxidative stress, followed by cell death [13,28,29,30,31,32]. However, the exact mechanism involved in these cytotoxic effects remains unclear. 

In the present study, for the first time, we compared the fungicidal activity of two biogenic Ag-NPs previously synthesized by our team using the novel cyanobacteria strains *Desertifilum* sp. IPPAS B-1220 and *Nostoc* sp. Bahar_M against *C. albicans* with that of AgNO_3_ (a precursor material for Ag-NP biofabrication). Moreover, we sought to determine how these biogenic Ag-NPs interfere with the fungal cell envelope at the ultrastructural level, as well as how they influence *C. albicans* enzyme activity, *Hwp1* and *CDR1* gene expression, and protein profile. These data will complement the existing information, and indicate that Ag-NPs synthesized by cyanobacteria may represent robust and potent fungicidal agents against *C. albicans*.

## 2. Materials and Methods

### 2.1. Materials

Silver nitrate (AgNO_3_), fungal culture medium, and enzyme activity assay kits (colorimetric; catalase [CAT], and glutathione peroxidase [GPx] were obtained from Sigma-Aldrich (St. Louis, MO, USA), while lactate dehydrogenase [LDH]) from Abcam (Cambridge, UK). PiBind resin was purchased from Expedeon (San Diego, CA, USA); TRIzol reagent was obtained from Life Technologies (Carlsbad, CA, USA); Maxima SYBR Green/Fluorescein qPCR Master Mix and the QuantiTects Reverse Transcription Kit was obtained from Qiagen (Germantown, MD, USA); and TriFast was purchased from Peqlab VWR (Radnor, PA, USA). The silver nanoparticles were previously synthesized by our team using *Desertifilum* sp. IPPAS B-1220 (D-SNPs) and *Nostoc* sp. Bahar_M (N-SNPs); their physicochemical properties were analyzed by ultraviolet–visible (UV–Vis) spectrophotometry, X-ray diffraction, Fourier-transform infrared (FTIR) spectroscopy, and scanning (SEM) and transmission electron microscopy (TEM). The D-SNP and N-SNP particle size ranged from 4.5 to 26 nm and 8.5 to 26.4 nm, respectively, with an average diameter of 14.7 ± 1.1 nm and 14.9 ± 0.5 nm, respectively [33,34]. 

### 2.2. Methods

#### 2.2.1. Preparation of NP suspensions

To prepare a stock solution of AgNO_3_, D-SNPs, and N-SNPs, 1.5 mg (powder form) of each treatment was separately mixed with 1 mL of distilled water.

#### 2.2.2. Fungal Cell Culture

*C. albicans* Ncpf 3179 was procured from Almery University Hospital (Alexandria, Egypt). A 10-µL aliquot of *C. albicans* stock (stored at −80 °C) was cultured in yeast–peptone–dextrose (YPD) broth (1% [*w*/*v*] yeast extract, 2% [*w*/*v*] peptone, and 2% [*w*/*v*] dextrose) overnight at 30 °C in an orbital shaker (200 rpm). An overnight culture of *C. albicans* (50 µL; 1 × 10^4^ CFU/mL) was streaked on YPD agar plates and incubated at 30 °C for 14 to 16 h [10]. Subsequently, 8-mm wells were created in all the plates for use in the agar diffusion assay.

#### 2.2.3. Agar Well Diffusion Assay 

The agar well diffusion method was employed to evaluate the biocidal potential of the three silver agents against *C. albicans*. For this, 100 µL of 1.5 mg/mL AgNO_3_, D-SNPs, N-SNPs, and 12.5 µg/mL of amphotericin B (positive control), as well as distilled H_2_O (negative control) were added to individual 8-mm wells in the plates. Then, the plates were incubated overnight at 30 °C and the inhibition zone diameter (IZD) in mm was estimated using a transparent ruler [13]. 

#### 2.2.4. Measurement of Minimum Inhibitory Concentration (MIC) and Minimum Fungicidal Concentration (MFC)

MIC represents the lowest concentration of antimicrobial drug at which 90% of the growth of fungal cells is inhibited, while MFC represents the lowest concentration of antimycotic agents at which 100% of fungal cells are killed [35]. The microdilution method was used to estimate the MIC and MFC of AgNO_3_, D-SNPs, and N-SNPs against *C. albicans* based on M27A2 Clinical and Laboratory Standards Institute guidelines. A fungal suspension (100 µL/well of 1 × 10^4^ CFU/mL) was seeded into each well of a 96-well plate, individually mixed with 100 µL of different concentrations of AgNO_3_, D-SNPs, and N-SNPs (2.4, 2.1, 1.8, 1.5, 1.2, 0.9, 0.6, and 0.3 mg/mL), and amphotericin B (100, 50, 25, 12.5, 6.25, 3.12, and 1.56 µg/mL) and incubated overnight at 30 °C. MIC values were estimated by comparing the fungal turbidity with that of a 0.5 McFarland standard medium using the naked eye. MFC values were estimated by choosing the transparent wells, which indicate that *Candida* growth was totally prevented after being treated with the three agents, then 10 µL of this fungal suspension was recultivated on drug-free YPD agar plates. Thereafter, the plates were incubated at 37 °C for 24 h. The completely clear plate devoid of any colony at the last dilution is its MFC [36].

#### 2.2.5. Analysis of LDH Activity

The effect of AgNO_3_, D-SNPs, and N-SNPs on *C. albicans* membrane integrity was evaluated by analyzing the LDH activity in *C. albicans* before and after the treatment with 1.5 mg/mL AgNO_3_, D-SNPs or N-SNPs for 24 h at 30 °C. Briefly, the fungal cultures were centrifuged at 5000 rpm for 10 min at 4 °C. The resultant pellets were rinsed twice with a phosphate buffer saline (PBS) and then mixed with the LDH reaction solution under gentle shaking for 30 min at ambient temperature, following the manufacturer’s instructions. The optical density (OD) of the samples was measured at 490 nm [37].

#### 2.2.6. Estimation of ATPase Activity

The potential of the three treatments to negatively affect the metabolic activity of C. *albicans* was evaluated by measuring adenosine triphosphatase (ATPase) levels in C. *albicans* supernatants before and after exposure to 1.5 mg/mL AgNO_3_, D-SNPs or N-SNPs for 24 h utilizing a colorimetric ATPase assay, according to the manufacturer’s recommendations, as previously described in [38]. In brief, the experiment was performed utilizing membrane preparations (40 to 90/100 μL) previously kept with PiBind resin to eliminate the free inorganic phosphate (Pi) that could negatively affect the ATPase assay results. The samples were preincubated for 10 min at 37 °C with three treatments (1.5 mg/mL) to evaluate the influences of these agents on the ATPase activity. The quantity of Pi liberated was estimated using an UV 2505 spectrophotometer (Thomas Scientific, NJ, USA) at A_650_. The assay calibration was performed using a standard range of Pi concentrations, and data were determined for a minimum of three independent assays.

#### 2.2.7. Estimation of Antioxidant Enzyme Activity

The ability of AgNO_3_, D-SNPs, and N-SNPs to induce oxidative stress in *C. albicans* cells was estimated by analyzing GPx and CAT activity in *C. albicans* supernatants using the corresponding kits, and following the manufacturers’ instructions. Fungal cells treated or not with 1.5 mg/mL of each agent for 24 h at 30 °C were collected by centrifugation at 10,000 rpm for 5 min at 4 °C, washed at least twice with PBS, and then lysed using a sonicator. GPx and CAT activities were evaluated by measuring the OD of each sample at 340 and 240 nm, respectively [39]. 

#### 2.2.8. Visualization of *C. albicans* Cells by TEM

The toxic effects of AgNO_3_, D-SNPs, and N-SNPs on *C. albicans* morphology were assessed by TEM. Briefly, *C. albicans* cells treated or not with 1.5 mg/mL of each of the three treatments for 24 h at 30 °C were collected by centrifugation at 3500 rpm for 10 min. The resultant pellets were rinsed in PBS at least three times to remove any excess nanoparticles, fixed in ice-cold 4F1G (a mixture of 4% formaldehyde and 1% glutaraldehyde) in PBS for 2 h, stained with 1% osmium tetroxide (OsO_4_) for 2 h at ambient temperature, washed with PBS to eliminate excess OsO_4_, and dehydrated by immersion in 25%, 50%, 75%, 95%, and 100% ethanol. The dehydrated samples were infiltrated with propylene oxide and embedded in an Araldite Epon mixture. The specimens were cut into ultrathin (70 nm) sections on a LKB Ultramicrotome using a glass knife, double-stained with 2% uranyl acetate and lead citrate, and loaded onto 200-mesh copper grids for visualization under a JEOL 100 CX TEM (JEOL, Tokyo, Japan) operating at 80 kV [40]. The *C. albicans* cell size (before and after the treatment with the three silver agents) and NP size distribution in at least 10 TEM images were measured using the ImageJ software. 

#### 2.2.9. *Hwp1* and *CDR1* Gene Expression Analysis

The expression levels of the *Hwp1* and *CDR1* genes in *C. albicans* treated or not with 1.5 mg/mL AgNO_3_, D-SNPs, and N-SNPs for 24 h were analyzed by qRT-PCR. Total RNA was extracted from samples using the TRIzol reagent and reverse-transcribed using the QuantiTects Reverse Transcription Kit and random hexamer primers. The gDNA Wipeout Buffer was used to remove contaminating genomic DNA (gDNA). The resultant cDNA (30 ng) was used as a template for amplification using specific primer pairs (Table 1) at a final concentration of 300 nM. The qPCR was performed with SYBR Green/Fluorescein qPCR Master Mix in a Rotor-Gene Q instrument. Glyceraldehyde-3-phosphate dehydrogenase (*GAPDH*) was used as a housekeeping gene. The Rotor-Gene Q automatically compiled the data and determined the threshold cycle (Ct) value, which was normalized to the average Ct value of the housekeeping gene (ΔCt). The relative expression level of each gene was calculated as 2^−ΔCt^ [41,42].

#### 2.2.10. Sodium Dodecyl Sulfate–Polyacrylamide Gel Electrophoresis (SDS–PAGE)

The toxicity of AgNO_3_, D-SNPs, and N-SNPs (1.5 mg/mL) against *C. albicans* cellular proteins was examined in *C. albicans* treated or not with each of the three treatments for 24 h. The protein was extracted and purified using the TriFast reagent. Then, 10 µg of the purified protein was fractionated using an OmniPAGE Mini vertical electrophoresis unit with a Power Pro 5 power supply (Cleaver Scientific, Warwickshire, UK) on a precast gel (SERVAGel TG PRiME 10%; SERVA, Heidelberg, Germany). The gel was subsequently stained for 2 h with 0.1% Coomassie blue R-250 and de-stained with a solution of glacial acetic acid, methanol, and water (1:3:6). Data were analyzed using the TotalLab analysis software version 1.0.1 and a gel documentation system (GelDoc-It, UVP, Cambridge, UK) [13,44].

### 2.3. Statistical Analysis

All of the statistical analyses, including frequency distribution, descriptive analysis, and one-way analysis of variance (ANOVA), were performed using GraphPad Prism 8.3 (GraphPad Software Inc., La Jolla, CA, USA). Data were collected from at least three independently repeated experiments and are presented as means ± standard error of the mean (SEM). *p*-values < 0.05 were considered significant. 

## 3. Results

### 3.1. The Anticandidal Effects of AgNO_3_ and the Biogenic Silver Nanoparticles

**Figure 1:**
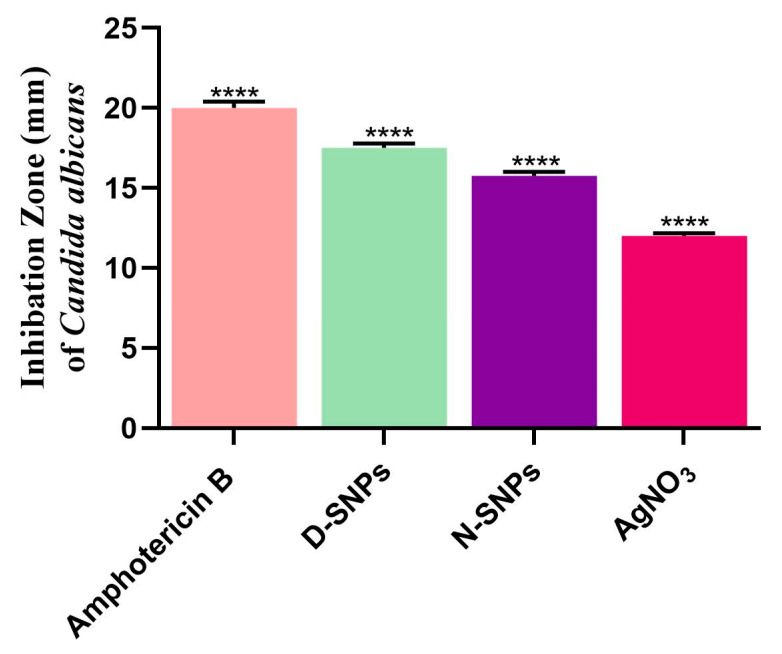
Fungicidal activity of amphotericin B, Ag-NPs fabricated by *Nostoc* sp. Bahar_M and *Desertifilum* sp. IPPAS B-1220 (N-SNPs and D-SNPs, respectively), and silver nitrate (AgNO_3_) against *Candida albicans*. Data were collected from at least three independent assays and are presented as means ± SEM. **** *p <* 0.0001 vs. untreated *C. albicans*.

The results of the agar well diffusion assay showed that AgNO_3_, D-SNPs, and N-SNPs exerted significant inhibitory activity against *C. albicans* (Figure 1). The highest IZ (20 ± 0.4) was seen with the amphotericin B (positive control) treatment. D-SNPs displayed the greatest inhibitory activity against *C. albicans*, with an IZD of 17.5 ± 0.3 mm compared with N-SNPs (IZD of 15.8 ± 0.3 mm) and AgNO_3_ (IZD of 12 ± 0.17 mm). Similar results were obtained in the serial dilution assay, namely, that *C. albicans* exhibited the same response to AgNO_3_, D-SNPs, N-SNPs, with MIC and MFC values of 1.2 and 1.5 mg/mL, respectively (Table 2).

### 3.2. The Influence of AgNO_3_ and the Biogenic D-SNPs and N-SNPs on C. albicans Enzyme Activity

**Figure 2:**
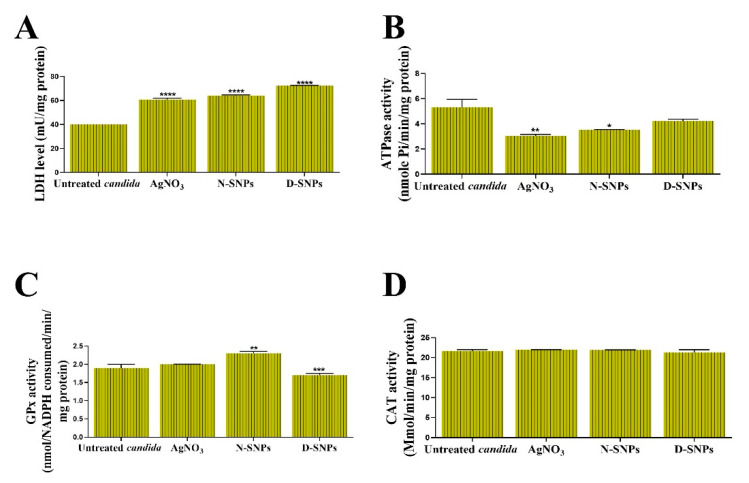
The influence of AgNO_3_, N-SNPs, and D-SNPs on *Candida albicans* enzyme activity. (**A**) Lactate dehydrogenase (LDH), (**B**) adenosine triphosphatase (ATPase), (**C**) glutathione peroxidase (GPx), and (**D**) catalase (CAT). Data were collected from at least three independent assays and are presented as means ± SEM. **** *p* < 0.0001, *** *p* = 0.0001, ** *p* < 0.001, and * *p* = 0.01 vs. untreated *C. albicans*.

The toxic influence of AgNO_3_, D-SNPs, and N-SNPs on *C. albicans* membrane integrity was evaluated by analyzing the LDH level in *C. albicans* culture supernatants 24 h post-treatment. The results showed that LDH levels were significantly higher in *C. albicans* cells treated with the three therapeutic agents than the untreated cells. The greatest increase in LDH levels was seen with the D-SNPs (Figure 2A).

The ability of AgNO_3_, D-SNPs, and N-SNPs to enhance metabolic toxicity in *C. albicans* cells was examined by measuring their ATPase activity. The results showed that, compared with the untreated controls, AgNO_3_ and N-SNPs induced a significant decline in ATPase levels. However, D-SNPs caused an insignificant decline in ATPase levels. Intriguingly, the greatest decrease in ATPase levels was recorded for AgNO_3_, followed by N-SNPs and then D-SNPs (Figure 2B). 

The capacity of AgNO_3_, D-SNPs, and N-SNPs to enhance oxidative stress in *C. albicans* was evaluated by measuring the levels of the antioxidant enzymes GPx and CAT in *C. albicans* culture supernatants. The results showed that GPx levels were similar between the untreated *C. albicans* cells and those treated with AgNO_3_. However, compared with the untreated controls, GPx levels were significantly higher in *C. albicans* cells exposed to N-SNPs, but significantly lower in those treated with D-SNPs. Furthermore, of the tested antifungal agents, N-SNPs induced the greatest increase in GPx levels in *C. albicans.* Interestingly, the AgNO_3_, D-SNPs, and N-SNPs treatment did not affect the CAT levels (Figure 2C,D).

### 3.3. Morphological Changes in C. albicans Cells Caused by AgNO_3_, D-SNPs, and N-SNPs 

**Figure 3:**
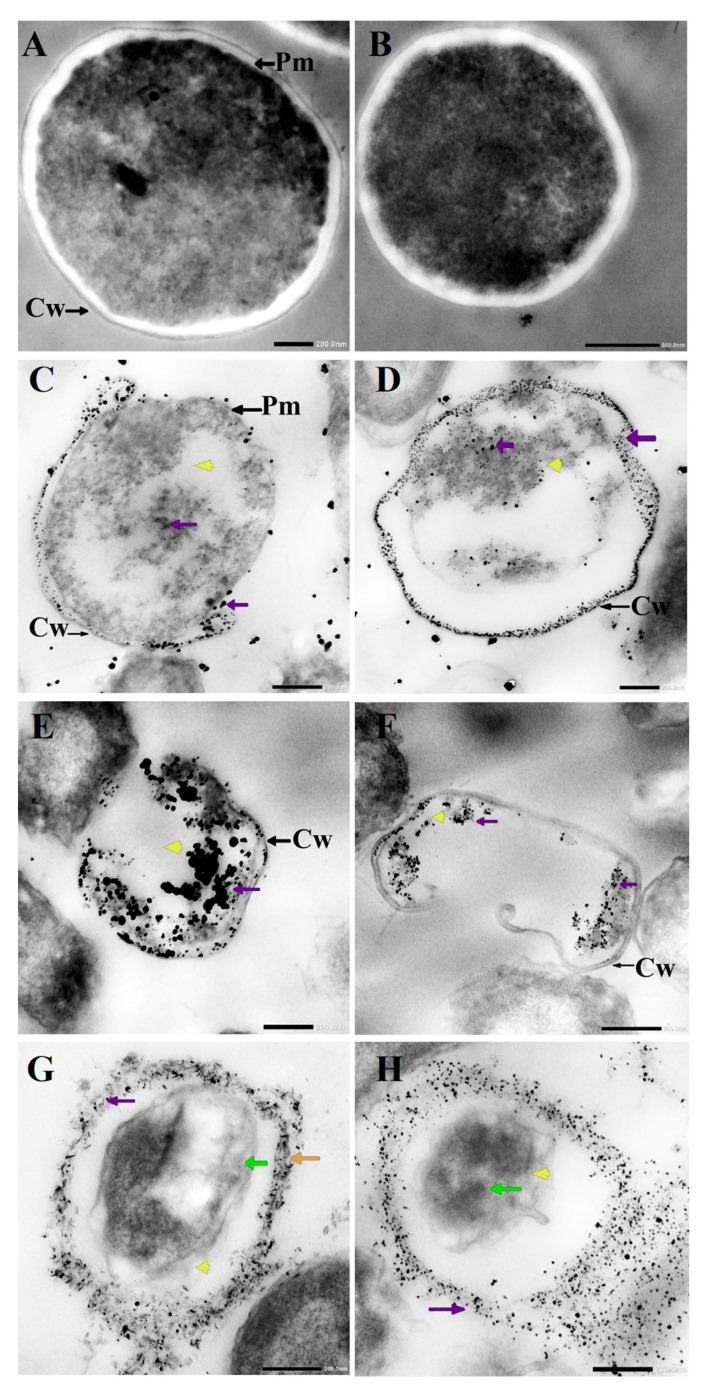
TEM micrographs of untreated *Candida albicans* exhibiting an intact cell wall (Cw) and intact plasma membranes (Pm) (**A**,**B**). TEM micrographs of *C. albicans* treated with 1.5 mg/mL AgNO_3_ showing the disruption of the cell wall (Cw) and folded membranes, the detachment of cellular membranes (Pm) around the cytoplasmic matrix, moderate cytoplasmic dissolution (yellow arrowhead), and deposition of dark spherical granules believed to be Ag-NPs synthesized by *C. albicans* itself (violet arrow) (**C**,**D**). TEM micrographs of *C. albicans* treated with 1.5 mg/mL D-SNPs showing the degradation of the cell wall (Cw), shrinkage, cellular membranes with dark, dense spherical particles thought to be D-SNPs (violet arrow), and severe cytoplasmic lashing (yellow arrowhead) (**E**,**F**). TEM micrographs of *C. albicans* treated with 1.5 mg/mL N-SNPs showing cell envelope lysis (orange arrow), severe cytoplasm lashing (yellow arrowhead), nucleoagglutination (yellow arrow), and distribution of dark, dense, spherical particles thought to be D-SNPs (violet arrow). (**G**,**H**) Scale bars: 200 and 500 nm.

**Figure 4:**
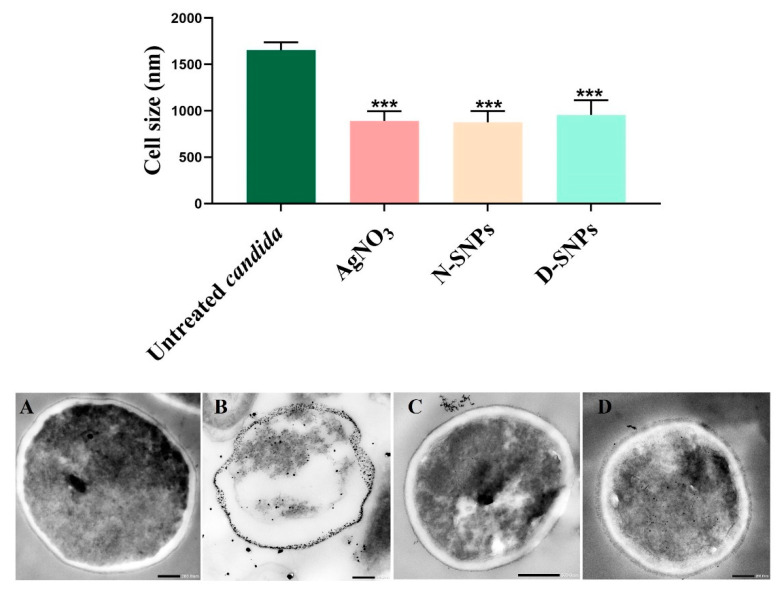
Bar graph and TEM micrographs depicting the diameter of *Candida albicans* cells (nm) before (**A**) and after AgNO_3_ (**B**), N-SNP (**C**), and D-SNP (**D**) treatment. Scale bars: 200 nm in A, B, and D; and 500 nm in C. Data were collected from at least three independent assays and are presented as means ± SEM. *** *p* < 0.0001 vs. untreated *C. albicans*.

**Figure 5:**
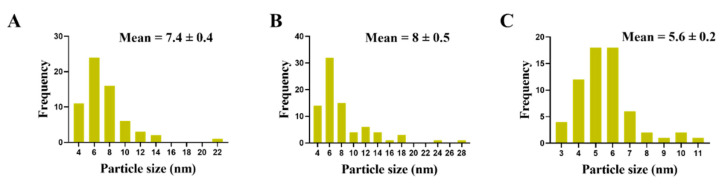
Particle size of Ag-NPs in *Candida albicans* cells treated with AgNO_3_ (**A**), D-SNPs (**B**) or N-SNPs (**C**).

TEM was used to determine the ultrastructural changes in *C. albicans* resulting from exposure to 1.5 mg/mL AgNO_3_, D-SNPs, and N-SNPs. The TEM micrograph analysis revealed that the untreated *C. albicans* presented typical cellular structures with a conserved and intact layered cell wall and distinctive cytoplasmic membranes (Figure 3A,B). In contrast, fungal cells exposed to AgNO_3_, D-SNPs or N-SNPs lost their distinctive morphological appearance and appeared to undergo lysis (Figure 3C–H). The treatment with AgNO_3_ induced the disintegration of fungal cells, disruption of the cell wall and cell membranes, separation of the cytoplasmic membrane from the cell wall, and cytoplasmic dissolution (Figure 3C,D). Additionally, the AgNO_3_ treatment led to a significant decrease in cell size (Figure 4), while dark, electron-dense spherical granules could be seen on the cell wall and membrane, as well as inside the fungal cells (Figure 3C,D). These granules ranged in size from 4 to 22 nm and had an average diameter of 7.4 ± 0.4 nm, suggesting that they were Ag-NPs resulting from the reduction of silver nitrate by *C. albicans* itself (Figure 5A). 

**Figure 6:**
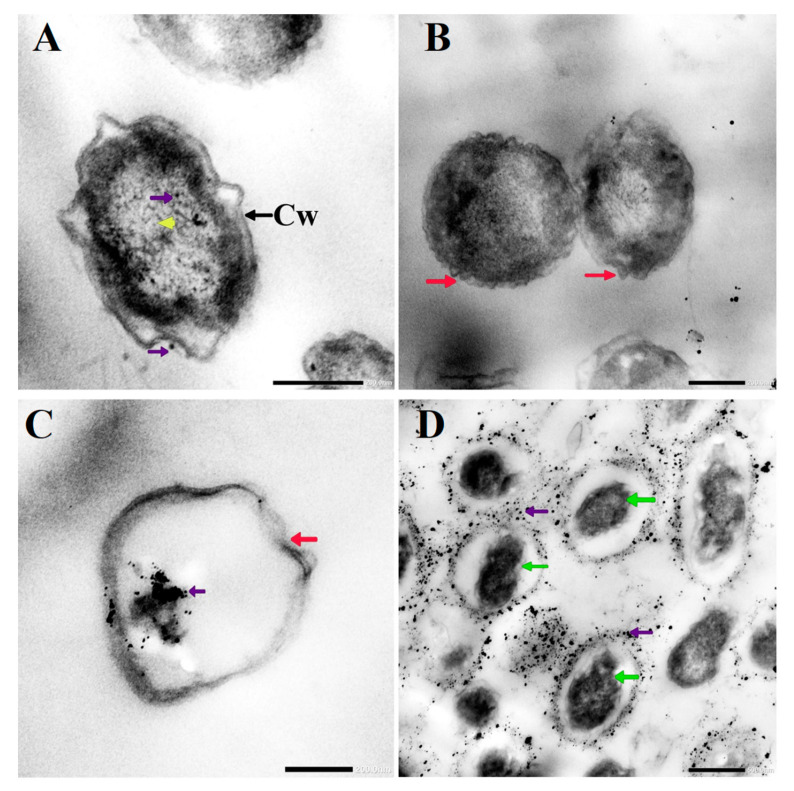
TEM micrographs of *Candida albicans* exposed to D-SNPs (**A**,**B**) and N-SNPs (**C**,**D**) showing the irregular cell wall (Cw), folded membrane (red arrow), cytoplasmic lashing (yellow arrowhead), nucleoagglutination (green arrow), and NP distribution inside and outside the cells (violet arrow).

Similarly, the D-SNP treatment triggered severe damage to the cell wall and plasma membranes. The D-SNP-treated *C. albicans* cells had thinner walls compared with the untreated cells, and underwent lysis of the cell envelope, in which layers within the cell wall were difficult to distinguish, and displayed severe cytoplasmic lysis (Figure 3E,F). Moreover, numerous pores, holes, and folds could be seen in the *C. albicans* cell envelope (Figure 6A,B). Furthermore, the D-SNP treatment resulted in a significant reduction in the cellular size of fungal cells when compared with that in the untreated cells (Figure 4). The D-SNPs were observed to surround the cell wall and also intensively agglomerate inside the fungal cells, displaying a size range of 4 to 28 nm and a diameter of 8 ± 0.5 nm (Figure 5B). Intriguingly, the N-SNPs caused extensive cell wall degradation, cytoplasmic membrane disruption, and agglutination of genetic material in *C. albicans* cells (Figure 3G,H). Moreover, the N-SNP-treated *C. albicans* cells had irregular, folded, and degraded cell walls (Figure 6C,D). Compared with the untreated fungal cells, the N-SNP treatment resulted in a significant reduction in cell size. The noteworthy drop in cell diameter of *C. albicans* was reported in the N-SNP treatment compared with the AgNO_3_ and D-SNP treatments (Figure 4). The N-SNPs were found to be extensively distributed at the cell borders, with a few being detected inside the fungal cells (Figure 3G,H and Figure 6C,D). These NPs had a size range of 3 to 11 nm and an average diameter of 5.6 ± 0.2 nm (Figure 5C). In summary, all of the treatments used in this study caused morphological alterations in *C. albicans* cells. However, the acutest changes were recorded with the D-SNP and N-SNP treatments. 

### 3.4. The qRT-PCR

**Figure 7:**
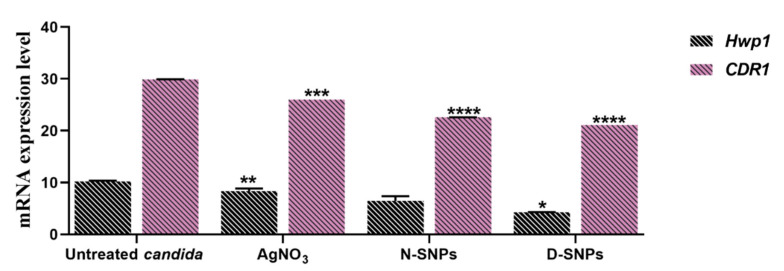
The mRNA expression levels of the *Hwp1* and *CDR1* genes in *Candida albicans* treated or not with AgNO_3_, N-SNPs, and D-SNPs. Data were collected from at least three independent assays and are presented as means ± SEM. **** *p* < 0.0001, *** *p* = 0.0001, ** *p* < 0.001, and * *p* = 0.01 vs. untreated *C. albicans*.

The effects of 1.5 mg/mL AgNO_3_, D-SNPs, and N-SNPs on *Hwp1* and *CDR1* gene expression in *C. albicans* were evaluated using qRT-PCR. The results revealed that, compared with untreated *C. albicans* cells, the expression of the *Hwp1* and *CDR1* genes was significantly downregulated in AgNO_3_- and D-SNP-treated cells. Meanwhile, the N-SNP treatment led to an insignificant downregulation of *Hwp1* gene expression and a significant downregulation of *CDR1* gene expression. Notably, among the three treatments, D-SNPs induced the most significant reduction in *Hwp1* and *CDR1* expression levels (Figure 7).

### 3.5. SDS–PAGE

**Figure 8:**
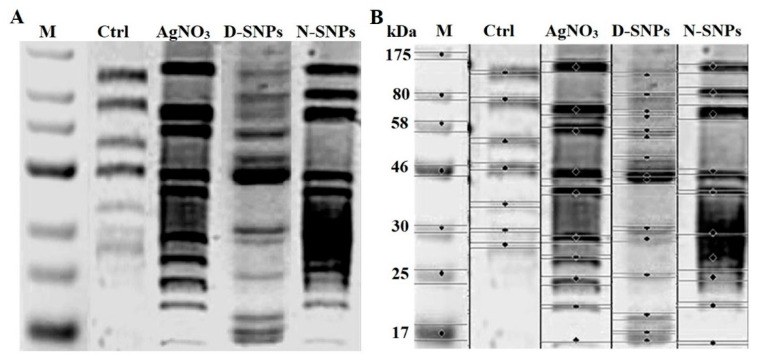
SDS–PAGE analysis of cellular proteins of *Candida albicans* (1 × 10^4^ CFU/mL) before and after exposure to 1.5 mg/mL AgNO_3_, D-SNPs, and N-SNPs for 24 h (**A**) and computerized analysis of protein band intensities (**B**). M: Marker; Ctrl: Untreated *C. albicans*.

An SDS–PAGE assay was used to assess the effect of 1.5 mg/mL AgNO_3_, D-SNPs, and N-SNPs on the protein profile of *C. albicans.* The protein pattern of *C. albicans* exposed to AgNO_3_ and N-SNPs was composed of protein bands with lower molecular weight compared with those of untreated and treated *C. albicans* with D-SNPs (Figure 8). However, more protein bands were detected for fungi treated with D-SNPs (15 bands) than for fungi treated with AgNO_3_ or N-SNPs (10 bands) or untreated fungi (7 bands) (Table S1).

## 4. Discussion

The results of the agar well-diffusion and serial dilution assays demonstrated that AgNO_3_, N-SNPs, and D-SNPs have potent inhibitory activity against *C. albicans*. However, among the three therapeutic agents, D-SNPs exerted the strongest antifungal effects against *C. albicans* relative to the untreated controls. Although the average size of the initial cluster of D-SNPs (14.7 ± 1.1 nm) was similar to that of N-SNPs (14.9 ± 0.5), the IZD against *C. albicans* was larger with the D-SNP treatment than with the N-SNP treatment. The differences in IZD values could be attributed to the differing nano/cell interface patterns resulting from differences in functional groups that surrounded the Ag-NPs, which are derived from different cyanobacterial strains [18,34]. This agrees with the results of our previous studies showing that the surface areas of N-SNPs and D-SNPs have different functional groups. For instance, N-SNPs have functional groups corresponding to aromatic compounds and proteins with FTIR spectral peaks at 1119.01, 1397.07, 1632.35, 1777.14, 2114.44, 2946.98, 3460.32, and 842.59 cm^−1^, corresponding to C-O secondary alcohol, O-H carboxylic acid, N-H amine, C=O anhydride or vinyl/phenyl ester, N=C=S isothiocyanate, O-H carboxylic acid or N-H amine salt, O-H alcohol, and C=C alkene, respectively. These results indicated that aromatic compounds and proteins might represent the reducing and stabilizing ligand of Ag-NPs during the biofabrication process [33]. However, D-SNPs have functional groups related to proteins and polysaccharides with FTIR spectral peaks at 601.81, 1042.35, 1626.05, 2353.23, and 3453.72 cm^−1^, corresponding to C-Br compounds, C-N amines of proteins and C-O vinyl ethers, N-H amine bonds of proteins and C=C alkenes, CΞC alkyne, and O-H polysaccharide and N-H amine bonds of proteins, respectively. Proteins and polysaccharides were reported to have a pivotal role in the reduction and stabilization of Ag-NPs [34]. Meanwhile, the two silver NPs showed a greater inhibitory effect than AgNO_3_ against *C. albicans*, which could be explained by the physicochemical properties of the NPs, including their smaller size, surface area, stability, and surface chemistry, which might enable greater surface contact with microbial membranes [45]. Akter et al. reported that the physicochemical properties of NPs, including their shape, size, stabilizing agent, and surface structure, can influence their antimicrobial potential [46]. Meanwhile, the synergistic effect of Ag-NPs synthesized by the fungus *Monascus purpureus* and pure *M. purpureus* extract was shown to significantly inhibit *C. albicans* growth, with an IZD of 16.7 ± 0.25, whereas using the *M. purpureus* extract alone resulted in an IZD of 10.1 ± 0.58 [47]. The authors reported that the Ag-NPs could enhance the inhibitory activity of fungal extracts by 1.73-fold compared with when the fungal extract was used alone. 

LDH is a cytosolic enzyme that is released from cells when their membrane is ruptured [48]. The results of the LDH activity assay revealed that all of the three tested treatments increased the levels of LDH released by *C. albicans* cells, suggesting that AgNO_3_, N-SNPs, and D-SNPs can all disrupt the membrane structure of *C. albicans* cells and increase their permeability. Korshed et al. demonstrated that the treatment with laser-generated Ag-NPs could dose-dependently increase the amount of LDH released by *Escherichia coli*, indicative of their potential to disrupt membrane integrity, thereby inducing bacterial cell death [49]. A recent report demonstrated that both Ag-NPs synthesized by *Nostoc* sp. Bahar_M and AgNO_3_ can increase the levels of LDH in *E. coli*, methicillin-resistant *Staphylococcus aureus*, *Salmonella typhimurium*, and *Streptococcus mutans* culture supernatants [27]. Similarly, Lange et al. found that the treatment with chemically synthesized Ag-NPs, copper (Cu)-NPs, and an Ag-Cu-nanocomplex promoted significant increases in the amount of LDH released by *Streptococcus agalactiae*, *Streptococcus dysgalactiae*, *Enterococcus faecalis*, *S. aureus*, *Salmonella Enteritidis*, *E. coli*, *Enterobacter cloacae*, and *C. albicans* cells.

In the present study, the greatest increase in LDH levels was associated with the D-SNP treatment, indicating that these NPs exert greater toxic effects on the *C. albicans* cell envelope (cell wall and membranes) than N-SNPs or AgNO_3_. This result could be attributed to differences in surface chemistry due to the different cyanobacterial compounds (biocoat) surrounding the D-SNPs and/or their smaller size, which would enable them to easily disrupt the integrity of the cell wall and penetrate the cells, resulting in cellular damage [16,46,50]. 

ATPases are enzymes that catalyze the hydrolysis of a phosphate bond in adenosine triphosphate (ATP), resulting in the release of Pi and the formation of adenosine diphosphate (ADP) [51]. ATPases have a pivotal role in fungal cell growth, pathogenicity, nutrient uptake, and pH regulation [52,53]. The results of the assay for the ATPase activity in culture supernatants of *C. albicans* cells treated with AgNO_3_, N-SNPs or D-SNPs for 24 h demonstrated that all of the three tested antifungal agents reduced ATPase levels in the fungal cells. These findings could be explained by the potential ability of the three treatments to directly promote a metabolic imbalance by interacting with the ATPase enzyme and/or indirectly by enhancing oxidative stress, leading to ATPase denaturation and dysfunction [13,27]. Exposure to chemically synthesized gold-NPs resulted in metabolic toxicity in *E. coli* via interfering with ATPase [54]. Based on the proteomic analysis of microbial cells treated with Ag-NPs, Dakal et al. suggested that Ag-NPs induce the accumulation of immature membrane precursor proteins, leading to the destabilization of the outer membrane of *E. coli*. ATPase translocation to the cell membrane requires energy from proton motive forces and ATP. Consequently, the accumulation of this protein could be attributed to the dissipation of proton motive forces and a reduction in cellular ATP levels, the latter perhaps due to leakage or inhibition of ATP synthesis [55]. 

Interestingly, AgNO_3_ was the fungicidal agent that caused the greatest reduction in ATPase levels, likely due to microbial heavy metal resistance during heavy metal detoxification [56]. 

ROS generation is mitigated by the antioxidative defense system. GPx and CAT are critical antioxidant molecules with roles in ROS metabolism and clearance [57]. Here, we found that the AgNO_3_ treatment did not lead to a significant change in the activity of these antioxidant enzymes, suggesting that the lethal effects of AgNO_3_ against *C. albicans* could be mediated through the disruption of the cell envelope and not via enhancing oxidative stress. Moreover, GPx levels were increased in *C. albicans* cells treated with N-SNPs and reduced in cells exposed to D-SNPs. These data could be explained by the capacity of both types of NP to induce oxidative stress via enhancing ROS production. We suggest that the different GPx-related responses between the N-SNP and D-SNP treatments were associated with fungal cell responses. For instance, in the case of N-SNPs, fungal cells would still be protecting themselves from N-SNPs via increasing GPx production to eliminate accumulated ROS. In contrast, D-SNPs could cause extensive damage to the structure of fungal cells, resulting in the loss of cellular functions and, consequently, their ability to detoxify Ag-NPs and withstand the intense NP-induced oxidative stress, leading to a drop in GPx activity [13,58]. Intriguingly, no significant changes in CAT levels were observed in *C. albicans* following the treatment with these two antifungal nanoagents, suggesting that resistance to NPs in *C. albicans* was mediated by the modulation of GPx expression, but not that of CAT. Similarly, Jiang et al. reported that, when treated with Ag-NPs (6 and 20 nm), the aquatic plant *Spirodela polyrhiza* showed a dose-dependent increase in superoxide dismutase activity, whereas that of CAT was unaffected [59]. Dong et al. examined the influence of Ag-NP size (10 ± 5, 30 ± 5, 60 ± 5, and 90 ± 5 nm) on their antibacterial potential and found that the smaller size was correlated with greater antibacterial activity [60]. The authors showed that the treatment with 10 nm of Ag-NPs induced the greatest ROS generation among the tested treatments, resulting in severe oxidative stress and extensive bacterial DNA and cell membrane damage. Meanwhile, Lee et al. compared the influence of two nanosized Ag-NPs (5 and 100 nm) and found that the 5 nm Ag-NPs, but not the 100 nm Ag-NPs, induced ROS formation in *C. albicans*. However, neither species affected the ROS levels in *S. cerevisiae*. The authors suggested that the mechanism involved in the fungicidal activity of Ag-NPs is dependent on the fungal species and response, and can be either dependent or independent of ROS generation [61]. 

The TEM results showed that D-SNPs, N-SNPs, and AgNO_3_ all triggered significant ultrastructural changes in *C. albicans* cells. These alterations could be categorized into two distinct patterns. The first involves alterations in the cellular envelope, including irregularity, disruption of the fungal cell wall and membranes, formation of folds and pores in the membranes, multilayered membrane shrinkage, and the detachment of the cellular membrane from the cytoplasmic matrix. These observations are in line with the results of the LDH analysis, in which D-SNPs, N-SNPs, and AgNO_3_ were shown to stimulate a significant increase in LDH activity in *C. albicans*, suggesting that the three treatments negatively affect fungal membrane integrity and permeability. These morphological alterations may occur via direct interaction between the Ag-NPs and cellular structure and their biomolecules, such as enzymes and proteins and/or the induction of oxidative stress via the NP-mediated promotion of ROS production, leading to fungal cell dysfunction, and, subsequently, the death of the cells [18,62]. Radhakrishnan et al. reported that Ag-NPs negatively affected the physical state of the cell envelope and the membrane fluidity of *C. albicans* cells [16]. Lara et al. reported that *C. albicans* treated with Ag-NPs (1 nm) were synthesized using a microwave-assisted method showing membrane disruption, pores and folds formation, increasing cell envelope thickness, and losing their typical appearance [10].

In the present study, we found that morphological changes in *C. albicans* varied according to the treatment, with the acutest changes being seen with the D-SNP treatment. This observation could be explained by the nature of the surface chemistry of the Ag-NPs, which was influenced by the biofunctional groups originating from the different cyanobacterial spp. Therefore, the greater antifungal activity of D-SNPs may derive from the properties of the biomolecules of *Desertifilum* sp., suggesting that they may have a pivotal role in enhancing the antifungal activity of Ag-NPs by endowing them with greater affinity for cell membranes, as well as improved cell-penetrating ability, compared with those of N-SNPs, the functional groups of which originated from *Nostoc* sp. [34]. Several studies have reported that *Nostoc* sp. extracts display weak or no antifungal activity against *C. albicans* [63,64].

Furthermore, the analysis of TEM micrographs of *C. albicans* cells treated with D-SNPs or N-SNPs showed the presence of dark, electron-dense particles distributed on the cell envelope (including the cell wall and membranes) and inside fungal cells. These particles were spherical and similar in size to the initial cluster of D-SNPs and N-SNPs. However, N-SNPs were found to be extensively attached to the cell envelope of fungal cells and relatively few were concentrated inside the cells, whereas the opposite was seen with the D-SNPs. One possible explanation for these observations may be related to differences in the ability of the two NP agents to penetrate the cell wall as a result of the presence of distinct biofunctional groups derived from different cyanobacterial strains. Another explanation may be associated with NP stability, whereby N-SNPs would tend to aggregate outside the cells, with only a few smaller-sized NPs entering the cells and causing only moderate morphological alterations relative to those caused by D-SNPs. Panáček et al. reported that stabilized Ag-NPs have greater inhibitory activity towards *Candida* spp. than non-stabilized Ag-NPs [65]. These potential strategies can be summarized in two steps, namely, electrostatic attraction between the NPs and the cell membrane are followed by the adsorption of NPs on the surfaces of fungal cells, leading to the formation of membrane folds and pores, as well as changes in membrane permeability and integrity. These effects are expected to facilitate the entry of the Ag-NPs into fungal cells, where they interact with cytoplasmic and nucleic contents, leading eventually to fungal cell death [18,27,40]. 

Intriguingly, *C. albicans* exposed to AgNO_3_ also exhibited dark, electron-dense particles with a size range of 4 to 22 nm and an average diameter of 7.4 ± 0.4 nm distributed on the cell wall and inside the fungal cells. We suggest that these are Ag-NPs synthesized by *C. albicans* itself as a defense strategy to detoxify the silver ions. However, the resultant NPs kill the fungal cells. This phenomenon was termed the “zombie effect”, in which microbes fabricate the NPs that kill them [66]. Rahimi et al. reported that *C. albicans* can biofabricate Ag-NPs. These NPs are spherical, range in size from 20–80 nm, and display significant biocidal activity against *E. coli* and *S. aureus* [67]. We propose that the Ag-NPs formed by *C. albicans* were responsible for the observed morphological alterations. Indeed, AgNO_3_-treated *C. albicans* cells exhibited similar changes to those resulting from the D-SNP and N-SNP treatments, including membrane disruption, the detachment of fungal membranes, and moderate cytoplasmic dissolution [30]. 

The second pattern of morphological alterations observed in *C. albicans* cells exposed to D-SNPs, N-SNPs or AgNO_3_ comprises changes in cytoplasmic and nuclear contents, including severe cytoplasmic dissolution and nucleoagglutination. These data suggest that the three treatments may induce fungal cell death either directly by interfering with cellular contents, such as nucleic acids and proteins or indirectly by enhancing ROS generation (for the D-SNP and N-SNP treatments only), leading to severe oxidative stress and, consequently, biomolecule denaturation and cell damage [16,18].

The cell wall is a pivotal structure for fungal pathogenicity, with important functions in adhesion, invasion, and morphological conversion. Cell wall damage influences the ability of fungal cells to form biofilms and invade host cells [68]. The hyphal cell wall protein *Hwp1* is a cell surface protein that is covalently linked to the cell wall glucan through a remnant of its glycosylphosphatidylinositol anchor. It mimics the host cell transglutaminase 1 substrate and produces tight attachments to epithelial cells that are required for oroesophageal candidiasis [69]. This protein is a key adhesion molecule important for *C. albicans* pathogenicity as well as its capacity to transition from the yeast to the hyphal form, which contributes to biofilm formation [70]. The qRT-PCR results demonstrated that D-SNPs, N-SNPs, and AgNO_3_ can downregulate *Hwp1* gene expression in *C. albicans*, which may be attributable to the membrane damage caused by the three treatments influencing hyphae formation and adhesion properties in *C. albicans*. The largest decrease in *Hwp1* gene expression occurred with the D-SNP treatment, suggesting that the potent fungicidal activity of D-SNPs includes the inhibition of biofilm formation. Aslani et al. reported that exposure to zinc oxide (ZnO)-NPs coated with chitosan-conjugated linoleic acid led to a significant reduction in *Hwp1* gene expression in *C. albicans*, suggestive of its ability to influence biofilm formation and control *Candida*-related infections [41].

Candida drug resistance 1 protein (*CDR1*) is one of the ABC transporters, transmembrane efflux pumps that mitigate the movement of small hydrophobic molecules out of the cells by utilizing ATP [71]. Therefore, it is considered as a multidrug resistance gene found in *C. albicans*, and is reported to be responsible for *C. albicans* resistance to azole [72]. In the present study, D-SNPs, N-SNPs, and AgNO_3_ all downregulated *CDR1* gene expression in *C. albicans*, possibly due to DNA structural disruption resulting from a direct interaction between DNA molecules and NPs and/or enhanced oxidative stress. However, the largest decrease in *CDR1* gene expression was observed in D-SNP-treated *C. albicans* cells. These results suggested that the NP-related decrease in the resistance of *C. albicans* to antimycotic medicines was mediated through the downregulation of the expression of drug resistance-related genes, and further suggest that D-SNPs may have the potential to enhance the therapeutic activity of many antifungal drugs against which *C. albicans* is resistant. Parsameher et al. reported that exposure to biogenic selenium NPs led to the downregulation of *CDR1* gene expression in fluconazole-resistant and -susceptible *C. albicans* isolates [42]. 

In the SDS–PAGE results, more bands were detected in the protein profile of fungi treated with D-SNPs (15 bands) than in that of untreated fungi (7 bands) or fungi treated with AgNO_3_ or N-SNPs (10 bands). These data indicated that the three tested antifungal agents promoted protein denaturation and degradation. However, the greatest negative impact on the protein profile of *C. albicans* was detected with the D-SNP treatment. In addition, this could be attributed to the unique physicochemical features of the D-SNPs, such as their small size and the nature of their surface chemistry. These properties may increase the affinity between the NPs and the thiol groups of proteins, leading to the protein chain unfolding and potentially resulting in protein modification and degradation. Furthermore, ROS produced through the NP treatment may also influence protein configuration and enhance protein degradation. Moreover, the appearance of new bands in the protein profiles of *C. albicans* treated with D-SNPs, N-SNPs, and AgNO_3_ compared with those of untreated *C. albicans* are suggestive of the production of new proteins by *C. albicans* as a defense mechanism against silver agents [13,73]. In a similar analysis, Tamiyakul et al. found that the *S. aureus* and *E. coli* cells treated with Ag-NPs functionalized with poly (4-styrenesulfonic acid-co-maleic acid) polymer showed more bands in their protein profiles than the corresponding untreated cells, and suggested that the Ag-NP treatment induced bacterial death through interfering with the metabolic function and cell wall, nucleic acid, and protein synthesis [74].

**Figure 9:**
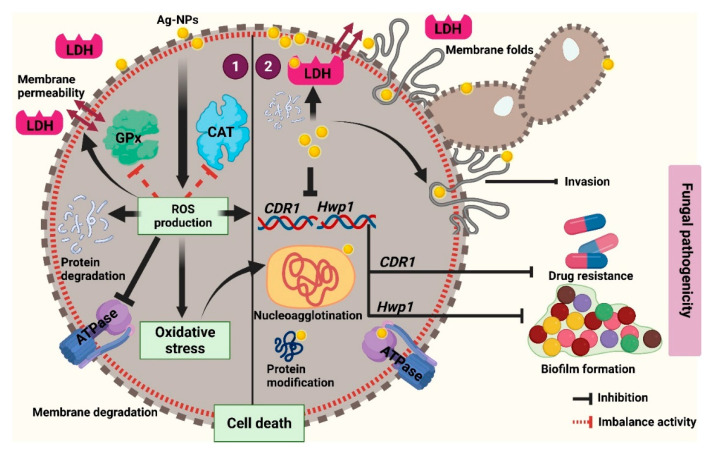
Schematic diagram illustrating the possible mechanism of D-SNPs and N-SNPs (Ag-NPs) against *C. albicans*.

Combined, our data suggest that D-SNPs and N-SNPs may exert their fungicidal effects by directly interfering with the cell envelope, resulting in the disruption of membrane permeability and integrity, which affects transport through cytoplasmic membranes and finally leads to fungal cell death. This membrane damage further leads to the loss of *C. albicans* pathogenicity, as well as that of its invasive and adhesive abilities, resulting in fungal death. Our results further suggest that D-SNPs and N-SNPs can enhance ROS production in *C. albicans* cells, leading to the induction of oxidative stress and the loss of metabolic balance and damage to cell structures and biomolecules, such as enzymes, proteins, and DNA, finally resulting in bacterial cell death. Additionally, we found that D-SNPs exerted greater toxicity than N-SNPs or silver nitrate against *C. albicans* cells, which we suggest could be attributed to the presence of *Desertifilum* biomolecules that surround the SNPs, which increases their stability, prevents their agglomeration, and improves their cell wall disruptive effects, thereby enhancing their antifungal activity [13,34]. In contrast, the mechanism associated with the lethality of AgNO_3_ could be attributed to the direct effects of silver ions on the cell envelope, which induces cell wall and membrane damage, as well as an increase in membrane permeability, leading to apoptosis via directly interacting with biomolecules, such as enzymes, proteins, and DNA, and further leading to cellular dysfunction and eventually cell death [10,18] (Figure 9).

## 5. Conclusions

The findings of this study shed novel insights on the biocidal activity and the mechanism underlying the lethal effects of AgNO_3_ and two novel biogenic Ag-NPs—D-SNPs and N-SNPs—against *C. albicans*. The three treatments significantly inhibited candidal growth and increased LDH leakage, which was suggestive of fungal membrane disruption. Analysis of the antioxidant enzyme activity suggested that the resistance to NPs in *C. albicans* was mediated by the modulation of GPx expression, but not that of CAT. The three treatments led to a large decrease in ATPase activity, resulting in metabolic toxicity, ultrastructural alterations, protein degradation, and downregulation of *Hwp1* and *CDR1* gene expression, suggestive of their ability to negatively affect biofilm formation and the resistance potential of *C. albicans*. D-SNPs exerted the strongest fungicidal activity among the tested antifungal agents, suggesting that the surrounding biocoat derived from *Desertifilum* sp. can significantly enhance the D-SNP antifungal activities. Our results indicated that the lethal effects of D-SNPs and N-SNPs against *C. albicans* involved direct nano/cell interference through their interaction with cellular structures and/or indirect effects, which included enhancing ROS production, leading to severe oxidative stress and, consequently, biomolecule dysfunction, structural damage to cells, and, ultimately, apoptosis. Further studies are warranted to determine the inhibitory influence of D-SNPs and N-SNPs on other fungal strains, focusing on the mechanisms underlying the lethal effects of these NPs against fungal cells and the influence of cyanobacteria-derived functional groups surrounding the Ag-NPs on antifungal activities. Additionally, revealing the synergistic effects of N-SNPs and D-SNPs in combination with other antimycotic drugs is pivotal for evaluating the potential of these NPs to enhance the antimycotic drug activity against MDR microbes.

## Figures and Tables

**Table 1:** Primers used for qPCR.

Gene	Primers	Reference
*Hwp1*	F: GCT ACC ACT TCA GAA TCATCA TC R: GCA CCT TCA GTC GTA GAG GAC G	[43]
*CDR1*	F: GGTGCTAATATCCAATGTTGG R: GTAATGGTTCTCTTTCAGCTG	[42]

**Table 2:** Inhibition zone diameter (IZD), minimum inhibitory concentration (MIC), and minimum fungicidal concentration (MFC) of AgNO_3_, N-SNPs, and D-SNPs and amphotericin B against *Candida albicans*.

Fungi	Measurements	Treatments			
		AgNO_3_	N-SNPs	D-SNPs	Amphotericin B
*Candida albicans*	IZ (mm)	12 ± 0.17	15.8 ± 0.3	17.5 ± 0.3	20 ± 0.4
	MIC (mg/mL)	1.2	1.2	1.2	0.00625
	MFC (mg/mL)	1.5	1.5	1.5	0.0125
	MIC/MFC	0.8	0.8	0.8	0.5

## Data Availability

The data supporting this article are shown in Figure 1, Figure 2, Figure 3, Figure 4, Figure 5, Figure 6, Figure 7, Figure 8 and Figure 9 and three tables. The datasets analyzed in the present study are available from the corresponding author upon reasonable request.

## Supplementary Materials

The following are available online at https://www.mdpi.com/article/10.3390/pharmaceutics13101688/s1, Table S1: Number and intensity of bands in the protein profiles of C. albicans treated or not with 1.5 mg/mL AgNO3, N-SNPs, and D-SNPs.
