# Supplementary material for: Anticandidal Potential of Two Cyanobacteria-Synthesized Silver Nanoparticles: Effects on Growth, Cell Morphology, and Key Virulence Attributes of Candida albicans
Source: Pharmaceutics. 2021 Oct 15;13(10):1688. doi: 10.3390/pharmaceutics13101688 (PMC8539685; doi:10.3390/pharmaceutics13101688)
Supplement: Supplementary file 1 [file pharmaceutics-13-01688-s001.zip › pharmaceutics-1362551-supplementary.pdf]

pharmaceutics

Supplementary Materials: Anticandidal Potential of Two Cyanobacteria-Synthesized Silver Nanoparticles: Effects on Growth, Cell Morphology, and Key Virulence Attributes of *Candida albicans*

Reham Samir Hamida, Mohamed Abdelaal Ali, Doaa A. Goda and Alya Redhwan

Table S1. Number and intensity of bands in the protein profiles of *C. albicans* treated or not with 1.5 mg/mL $AgNO_3$, N-SNPs, and D-SNPs.

Control			$AgNO_3$			D-SNPs			N-SNPs		
Band No.	Lane%	MW (kDa)	Band No.	Lane%	MW (kDa)	Band No.	Lane%	MW (kDa)	Band No.	Lane%	MW (kDa)
1	0.82	122.884	1	1.93	136.418	1	1.08	87.891	1	2.16	136.418
2	1.35	73.210	2	1.83	64.003	2	0.67	62.713	2	1.67	80.000
3	1.44	52.290	3	14.19	55.070	3	1.43	58.063	3	3.33	61.001
4	1.82	46.438	4	1.89	39.284	4	3.82	58.039	4	1.4	45.546
5	1.66	35.992	5	1.59	28.242	5	1.29	58.039	5	0.7	39.284
6	1.13	29.338	6	1.04	26.270	6	0.96	56.840	6	9.84	28.755
7	1.64	27.389	7	2.83	24.498	7	0.62	47.534	7	7.9	26.132
			8	1.24	21.001	8	0.65	37.726	8	3.21	24.498
			9	14.73	16.059	9	0.93	35.658	9	1.96	21.001
			10	4.59	16.059	10	1.09	25.237	10	1.47	15.588
						11	3.09	23.056			
						12	1.33	23.056			
						13	2.20	19.451			
						14	1.57	17.584			
						15	7.86	16.781			
